# Risk Factors and Spatial Clusters of *Cryptosporidium* Infection among School-Age Children in a Rural Region of Eastern China

**DOI:** 10.3390/ijerph15050924

**Published:** 2018-05-06

**Authors:** Hao Zheng, Jianfeng He, Li Wang, Rong Zhang, Zhen Ding, Wenbiao Hu

**Affiliations:** 1 Department of Environmental Health, Jiangsu Provincial Center for Disease Control and Prevention, Nanjing 210009, China; zhenghao@jscdc.cn; 2 Department of Environmental Health, Jintan Center for Disease Control and Prevention, Changzhou 213200, China; dongtaihjf@163.com; 3 National Center for Rural Water Supply Technical Guidance, Chinese Center for Disease Control and Prevention, Beijing 102200, China; wangli@crwstc.org (L.W.); rzhang@crwstc.org (R.Z.); 4 School of Public Health and Social Work, Queensland University of Technology, Brisbane, Queensland 4059, Australia

**Keywords:** *Cryptosporidium*, risk factors, spatial clusters, children, rural, China

## Abstract

The epidemiological features of *Cryptosporidium* infection among school-age children in China still remain unclear. Hereby, a cross-sectional study of 1637 children aged 3–9 years was designed to investigate the risk factors and spatial clusters of *Cryptosporidium* infection in a rural region of Eastern China. Stool specimens collected from participants were examined using the auramine-phenol and modified acid-fast staining. Univariable and multivariable analyses were performed to identify the risk factors of *Cryptospordium* infection. The spatial clusters were analyzed by a discrete Poisson model using SaTScan software. Our results showed that the overall prevalence of *Cryptosporidium* infection was 11‰ in the research region. At the age of 3–6 years (odds ratios (OR) = 3.072, 95% confidence intervals (CI)*:* 1.001–9.427), not washing hands before eating and after defecation (OR = 3.003, 95% CI: 1.060–8.511) were recognized as risk factors. Furthermore, a high-risk spatial cluster (relative risk = 4.220, *p* = 0.025) was identified. These findings call for effective sustainable interventions including family and school-based hygienic education to reduce the prevalence of *Cryptosporidium* infection. Therefore, an early warning system based spatiotemporal models with risk factors is required to further improve the effectiveness and efficiency of cryptosporidiosis control in the future.

## 1. Introduction

The intestinal protozoan *Cryptosporidium* is considered to be a leading cause of the diarrheal diseases affecting humans globally. Since the first human cryptosporidiosis was found in 1976, it has been estimated that the prevalence of *Cryptosporidium* infection is <3% in industrialized regions and between 5 and ≥10% in developing regions [1,2,3]. Prior studies inferred that the high prevalence occurred more in socioeconomically-disadvantaged countries. The *Cryptosporidium* oocysts can remain infectious for several months owing to a high resistance to a variety of environmental pressures, such as disinfectants or pesticides. Individuals may be infected with oocysts directly or indirectly, through person-to-person transmission, consumption of contaminated food and water, or contact with contagious animals or domestic pets [4].

The clinical features of *Cryptosporidium* infection differ from asymptomatic status to a severe fatal outcome. Possible symptoms include diarrhea, vomiting, abdominal pain and fever. The severity of cryptosporidiosis depends on the age and immune status of individuals, varying from no symptoms or self-limiting diarrhea in immunocompetent persons to life-threatening outcomes in immunocompromised individuals [4]. Children are more vulnerable to *Cryptosporidium* infection than older age groups [5,6]. It is recognized that *Cryptosporidium* infection contributes to 30–50% of diarrhea mortality for children under five years in developing countries [2,7]. The results of a global multicenter study indicated that *Cryptosporidium* was second only to the rotavirus pathogen in leading to moderate-to-severe diarrhea among children younger than two years in developing countries [7]. Epidemiological studies revealed that early childhood cryptosporidiosis might be linked with malnutrition, growth impairment, and long-term cognitive function deficits [8,9,10].

In China, the first human case of cryptosporidiosis was confirmed in Nanjing, Jiangsu Province in 1987 [11]. After that, a number of studies concerning the epidemiology of cryptosporidiosis in different regions have been reported, estimating that the *Cryptosporidium* contributed to 1.40% to 5.66% of diarrhea episodes in China [12,13,14]. However, to date, cryptosporidiosis has not been involved in the national reported diseases systems. Furthermore, the examination of *Cryptosporidium* oocysts is not a routine check for the fecal samples of patients with diarrhea, which can lead to an underestimation of the prevalence. The main targeted groups of previous studies have been limited to patients with diarrhea or immunodeficiency diseases [13,14,15]. However, the epidemiology of *Cryptosporidium* infection among school-age children remains largely unclear, although children have been widely recognized as one of the most susceptible groups to *Cryptosporidium* infection. Furthermore, to facilitate the development of tailored and cost-effective cryptosporidiosis control and prevention strategies, it is desirable to delineate the spatial distribution of *Cryptosporidium* infection and to identify possible high-risk spatial clusters. This cross-sectional study attempted to fill these existing knowledge gaps. The specific objectives were two-fold: (a) to investigate the prevalence and possible risk factors of *Cryptosporidium* infection, and (b) to analyze spatial clusters of *Cryptosporidium* infection among school-age children in Jintan County, Jiangsu Province of Eastern China.

## 2. Materials and Methods

### 2.1. Study Area

**Figure 1:**
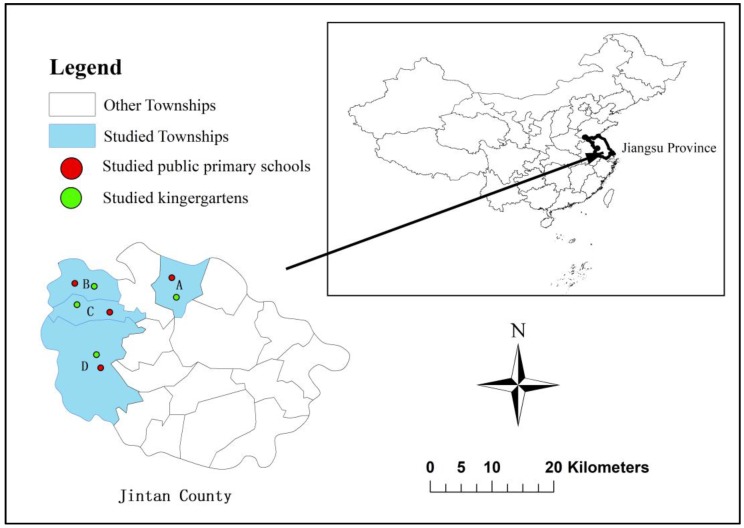
The study area, involving townships A, B, C, and D, studied public primary schools, and studied kindergartens in the study.

The cross-sectional study was carried out between July and October 2013 in a rural region of Jintan County, Jiangsu Province of Eastern China (Figure 1). Jintan County covers an area of 975 km^2^ and has a population of approximately 500,000 inhabitants, including 300,000 rural populations. Its coordinates are between 31.33° to 31.53° N and 119.17° to 119.44° E. The district is in the subtropical climate area. The average annual temperature is 15.3 °C (5–9 °C in winter, 29–32 °C in summer), and the annual average rainfall is between 900 and 1600 mm. Topographically, Western Jintan is high and Eastern Jintan is low. This district features relatively high-level socioeconomic status in China. Its economic income is mainly based on the production of basic grains, tourism, and commercial activities.

### 2.2. Data Collection

In 2013, there were 37 public primary schools and 41 kindergartens in Jintan County, and there were a total of 19,406 children aged 3 to 9 years in Jintan County (data provided by Jintan Education Bureau). In this study, four public primary schools and four kindergartens, located in four rural townships (Township A, B, C, and D), were randomly selected from all public primary schools and kindergartens in Jintan County (Figure 1). Using a stratified cluster sampling method, a total of 1651 children aged from 3 to 9 years were randomly selected from the above eight sites. Specifically, in the selected schools/kindergartens, children aged 3 to 9 years were identified first, and all these children were chosen as the participants in the investigation. To better perform the investigation, parents or guardians were first informed the purposes and procedures of the study, and written informed consent was provided to each of them. Further, a clean plastic container with a tag number was delivered to each parent or guardian to collect the stool samples of their children. When the written informed consent was completed, a self-administered questionnaire with demographic, environmental, and personal characteristics was filled in by the parents or guardians. The details of the questionnaire are summarized in Supplementary Table S1. Ultimately, only 1637 met the requirements, including written informed consent, questionnaires and fecal samples. There is no statistical difference between the included and excluded children in terms of age, gender and location.

Fecal samples (20–30 g) from each child were collected on the next day, and all feces were then sent to Jintan Center for Disease Control and Prevention (CDC) for determination and stored at 4 °C immediately. The oocysts of *Cryptosporidium* were examined using auramine-phenol and modified acid-fast staining [16]. In this method, *Cryptosporidium* oocysts were identified as pink to red on a blue background under fluorescence microscope. A stool was considered as positive if the oocysts fell with the size range of 4 to 6 µm. Each specimen was separately examined twice by two experienced technicians for quality control, and each run used one positive and two negative controls. Ten percent of the total examined smears were randomly selected, and then were sent to National Center for Rural Water Supply Technical Guidance, Chinese CDC for rechecking.

The study was performed in line with the Declaration of Helsinki. This study protocol was approved by the Ethics Committee of National Center for Rural Water Supply Technical Guidance, Chinese CDC (No. 201201). Children with *Cryptosporidium* infection were advised to go to the local hospital for further examination.

### 2.3. Descriptive and Risk Factors Analysis

Data were double input to EpiData software (version 3.0; The EpiData Association, Odense, Denmark). The prevalence of *Cryptosporidium* infection was expressed as permillage. The univariable analysis was performed by Pearson’s Chi-square test to identify the association between potential risk factors and *Cryptosporidium* infection, and the results were presented as crude odds ratios (cOR) with 95% confidence intervals (CI). Multivariable analysis was conducted to confirm the risk factors by logistic regression analysis (Enter), and the results were presented as adjusted odds ratios (aOR) with 95% CI. *Cryptosporidium* infection was used as the dependent variable and the demographic, environmental, and personal characteristics were taken as the independent variables. Factors with *p* values < 0.1 in the Pearson’s chi-square test were chosen as the independent variables in the logistic regression analysis. All statistical analyses were carried out using SPSS software (version 20.0; IBM SPSS Institute, Inc., Armonk, NY, USA). Differences were considered significant at *p* values ≤ 0.05.

### 2.4. Cluster Analysis and Spatial Visualization

To identify the high-risk spatial clusters, we employed a discrete Possion model using the SatScan software (version 9.4; Martin Kulldorff, Boston, MA, USA). The discrete Possion model requires cases and populations for a set of data districts. In this study, all cases were sorted into different villages according to the address of each case. Each village was marked using geographical coordinates on the map. Demographic information on Jintan County was provided by the local government. We input the details (number of cases, geographical coordinates, and populations) of the each village into Excel (version Office 2010; Microsoft Inc., Redmond, WA, USA), and then imported the file into the SatScan software. We chose a discrete Possion model with high rates to output the results of the spatial cluster analysis. We assumed the age group structures in each village were similar and used the total population of each village in the discrete Possion model. The results were shown using ArcGIS software (version 10.2; ESRI Inc., Redlands, CA, USA). The spatial distribution was estimated based on the observed cases in this study using the empirical Bayesian Kriging (EBK) method with the ArcGIS software. EBK is a geostatistical interpolation method that is widely used to estimate the spatial distribution of targeted diseases. In this method, the location (displayed by the latitude and longitude) of each case was marked on the map, and then we choose the EBK method to analyze the spatial distribution in Jintan County. Spatial visualization was shown on the map by ArcGIS.

## 3. Results

### 3.1. Characteristics of the Participants

A total of 1, 637 school-age children from four townships (A, B, C and D) were enrolled in the present study. These children were categorized into two groups by age: 3–6 years in the kindergarten stage (*n* = 863, 52.7%) and 7–9 years in the primary school stage (Grade 1 to Grade 3) (*n* = 774, 47.3%). The average age of the children was 6.4 ± 1.7 years.

In total, 864 (52.8%) were male and 773 (47.2%) were female. The sex ratios of male/female in 3–6 years and 7–9 years were 1.16 and 1.07, respectively. There was a higher proportion of participants from Township D (*n* = 741, 45.3%) compared with the other townships. The majority of the participants had access to piped water (97.4%, *n* = 1594) and household toilets (96.5%, *n* = 1580). Almost half of the participants (46.2%, *n* = 756) raised livestock or poultry. About a quarter of the children (25.4%, *n* = 416) reported that they had contact with pets. The general characteristics of the participants are presented in Table 1.

### 3.2. Prevalence and Risk Factors Associated with Cryptosporidium Infection

The results of the univariable and multivariable analyses of the risk factors associated with *Cryptosporidium* infection are presented in Table 2. Overall, 18 of 1637 fecal samples (11‰) were positive for *Cryptosporidium* infection. In terms of age and gender, the prevalence was 16‰ (14/863) among children aged 3–6 years and 5‰ (4/774) aged 7–9 years; 9‰ (8/864) for male and 13‰ (10/773) for female.

The results of the univariable analysis revealed that for the age group of 3–6 years (cOR = 3.175, 95% CI: 1.041–9.709, *p* = 0.032), location (F = 8.07, *p* = 0.031), and not washing hands before eating and after defecation (cOR = 3.088, 95% CI: 1.096–8.703, *p* = 0.025) were possible risk factors. In addition, the prevalence of infection in males was slightly lower than that of female children, although the difference was not statistically significant. The risks of *Cryptosporidium* infection did not differ according to the factors supply of piped water, presence of toilets in the household, raising livestock or poultry, contact with pets, boiling water before drinking, swimming in one month, travelling in six months, contact with water facilities in one month, and contact with a person with diarrhea in three months.

As gender may be a confounding factor, it was therefore controlled for in the multivariable logistic regression. Finally, the results of the multivariable logistic regression confirmed that participants aged 3–6 years were 3.072 times more likely to be infected with *Cryptosporidium* infection (aOR = 3.072, 95% CI: 1.001–9.427, *p* = 0.050). Children who did not wash their hands before eating or after defecation were 3.003 times more likely to be infected with *Cryptosporidium* infection (aOR *=* 3.003, 95% CI: 1.060–8.511, *p* = 0.039).

### 3.3. Cluster Analysis and Spatial Visualization

**Figure 2:**
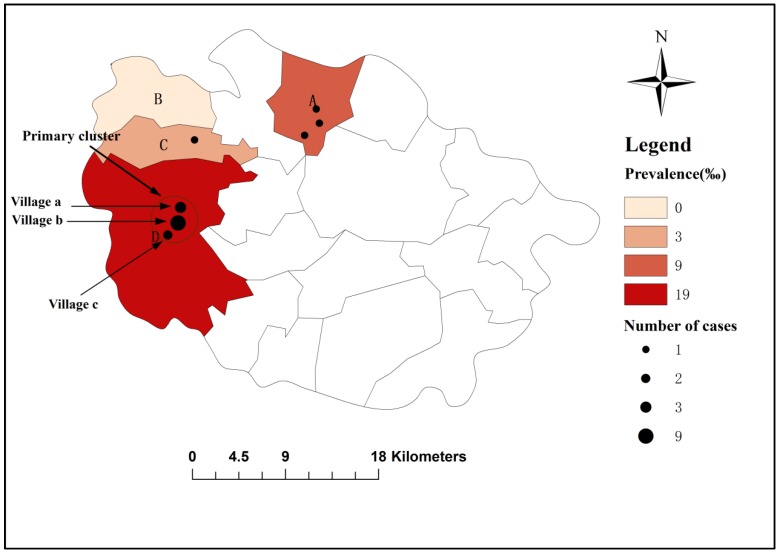
Spatial cluster and prevalence of *Cryptosporidium* infection in the study.

A total of 18 cases were confirmed in this study. The location of each case was marked on the map according to the address reported in the questionnaires. The total of 18 cases was distributed in seven villages and the number of cases from each village is shown on the map. As shown in Figure 2, the prevalence in Township A, C, and D was 9‰, 3‰, 19‰, respectively. No cases were found in Township B. The details of each village were input into the SatScan software to identify the spatial clusters. The results of a discrete Possion model analysis identified a high-risk primary cluster with a radius of 2.86 km in Township D (Relative Risk (RR) = 4.220, *p* = 0.025). This spatial cluster was composed of three villages (Village a, b, and c), and the observed and expected cases were 14 and 8.16 respectively. The number of cases in Village a, b, and c was 3, 9, and 2, respectively. Among the total of 14 cases in the cluster confirmed in Township D, 10 cases were from the same kindergarten, and two pairs of students are classmates. The details of the spatial cluster are shown in Table 3 and Figure 2.

**Figure 3:**
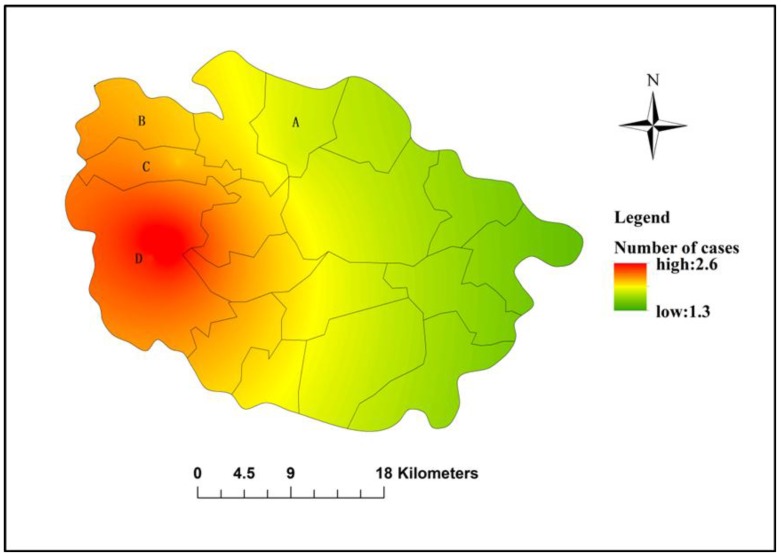
Spatial visualization of *Cryptosporidium* infection in Jintan County.

A spatial visualization of *Cryptosporidium* infection is delineated in Figure 3. It shows that the predicted cases increased from East Jintan to West Jintan gradually. The number of estimated cases increased from 1.3 to 2.6 in Jintan County.

### 3.4. Association of Cryptosporidium Infection with Gastrointestinal Symptoms

Most of the children (90.1%, 1475/1637) had no complaints about gastrointestinal signs or symptoms. Of the overall 18 cases, most cases (72.2%, 13/18) were asymptomatic. The remaining five cases reported diarrhea, and three cases were accompanied by nausea and vomiting symptoms. The prevalence of *Cryptosporidium* infection was significantly higher among children who had gastrointestinal symptoms than their asymptomatic counterparts (31‰ vs. 9‰, F = 4.656, *p* = 0.031).

## 4. Discussion

The present study investigated the prevalence and risk factors of *Cryptosporidium* infection among school-age children in Jintan County of Eastern China. Furthermore, the study identified a spatial cluster in Township D, and estimated the spatial visualization based on the observed cases. Previous studies revealed a possible association between increased temperature and precipitation and *Cryptosporidium* infection [17,18]. Accordingly, this investigation was chosen to be carried out during a rainy season with high temperature.

The prevalence of *Cryptosporidium* infection has been extensively reported in developing countries. Previous studies estimated that the prevalence ranged from 0.79 to 5.06% among the general population in China [12,16,19,20]. However, a recent community-based investigation reported that the prevalence was 12.0% among the general population (1–60 years) in a rural resource-limited region [21]. Al-Delaimy et al. found that the prevalence was 5.2% among school children in Malaysia [22]. Two studies performed in Ethiopia reported that the prevalence was 7.3% and 4.6% among school children, respectively [23,24]. Quihui-Cota et al. reported a high prevalence of 27% among children in Mexico [25]. The heterogeneity of the previously documented prevalence might to some extent be due to the socioeconomic level of the study site, the immune status of the targeted groups, and the sensitivity of the examining techniques. Our study suggested the overall prevalence was 11‰ among school-age children in the research region, which was lower than previous studies reported in other countries, and this might be attributable to two reasons. First, as discussed above, the site of this study features a relatively high socioeconomic level in China. Second, we detected the *Cryptosporidium* oocysts via the traditional microscopic examination, which may underestimate the true prevalence owing to low sensitivity.

Our finding indicated that children aged 3–6 years were more likely to be infected with *Cryptosporidium* infection than those aged 7–9 years. A similar result was found among infants and children in Xuzhou City, China [20]. This may be explained by the higher vulnerability of younger children and the relatively better personal hygiene awareness in older children. In our study, children between 3–6 years were still at kindergarten stage, and they may not receive as much hygienic education as those in primary schools. Besides, younger children may have a higher opportunistic exposure to contaminated sources of infection. The association between younger age and higher chance of *Cryptosporidium* infection was well-established in a number of studies in other countries [22,26,27].

In addition, we found that not washing hands before eating and after defecation was a significant risk factor for *Cryptosporidium* infection. This finding is also in line with similar studies reported in other regions [22,27,28,29]. Exposure to contaminated food and hand and skin penetration is an important transmission route for *Cryptosporidium* infection. Owing to a high resistance to the environment, infectious *Cryptosporidium* oocysts can survive for a long time. The absence of a good personal hygiene may facilitate transmission, and consequently increase the risks of *Cryptosporidium* infection. Despite the rapid development of the economy, family and school-based hygienic education is urgently needed for the children in this region.

No statistical difference was found between male and female children in terms of *Cryptosporidium* infection in our study. This is in agreement with observations in similar studies [22,23]. In addition, we did not observe any association between “supplied with piped water” and *Cryptosporidium* infection. Previous studies revealed that tap water was a risk factor for *Cryptosporidium* infection in other regions [25,30]. In our study, supplied drinking water covered most of the households. Furthermore, this region had a qualified drinking-water quality that meets the National Drinking Water Quality Standard. The drinking water quality surveillance also demonstrated the absence of *Cryptosporidium* oocysts. Finally, the majority of participants were used to boiling water before drinking in daily life. These reasons may remarkably decrease the risk of transmission via drinking water.

In this study, we identified a high-risk spatial cluster in Township D. In addition, children with *Cryptosporidium* infection also clustered in the same kindergarten (71.4%, 10/14), highlighting the importance of disease prevention in homes and schools. Besides, a very famous scenic location called Mao Mountain is mainly located in Township D. The scenic location attracts more than a million visitors every year. The intense mobility of persons, consequently, may increase the risk of transmission. Finally, high temperature during the rainy season with a mountainous topography generates a humid environment, which may increase the likelihood of infection. However, the reasons for the cluster still remain unclear in our study. Further studies considering the molecular techniques may help us to explore the exact reasons behind this in the future.

The results of the spatial visualization showed a high to low tendency of estimated cases from West to East of Jintan County. The results suggest that West Jintan County has a higher risk of infection than other parts. Therefore, this region may be taken as a priority site for future investigation. This finding is consistent with the results above. However, in the process of spatial analysis, we only considered the locations of the cases. Other potential factors such as socioeconomic and travelling information were not taken into account in the present study. Therefore, large-scale and comprehensive studies are required to explore the epidemiological features.

Our study also found that 72.2% (13/18) of *Cryptosporidium*-positive children were asymptomatic. A cohort study performed in Peru indicated that even if asymptomatic cryptosporidiosis has a less severe effect on weight gain than symptomatic counterparts, it still has a more adverse effect on child growth [31]. Symptomless children can act as potential sources of infection by excreting contagious oocysts in feces. Intimate individuals at home or school may cause fecal–oral route infection. In view of the strong resistance of *Cryptosporidium* oocysts to the environment, the infection can last for a long time. In light of the ubiquity of asymptomatic cryptosporidiosis, more attention is needed from public health officials.

To the best of our knowledge, this is the first cross-sectional study of school-age children in Jintan County. The spatial cluster analysis suggested a potentially high-risk cluster in Township D requiring further study. We found that “children aged 3–6 years” and “not washing hands before eating and after defecation” significantly increased the likelihood of infection compared with other factors. Intervention measures such as family and school-based hygienic education needs to be strengthened among the susceptible population in this region.

There are several limitations in this study. Firstly, owing to limited of resources, only one single fecal sample was collected from each child, although three consecutive samples would have been more ideal. Secondly, the prevalence might be underestimated due to the low sensitivity of the stool sample examination technique. However, the auramine-phenol and modified acid-fast staining method used in the study is more sensitive than modified acid-fast staining alone [19]. Furthermore, we took strict quality control measures (the details were described in the Methods section) to overcome this limitation. Finally, this study was restricted to four rural townships, which hardly represents the overall status of the whole region. The study had relatively little sampling, so the distribution of sampling could be biased if interpolating the whole county. In future research, more evenly distributed sampling sites are required to confirm these results.

## 5. Conclusions

Despite the relatively low prevalence reported in this study, *Cryptosporidium* infection still remains an important public health issue in Jintan County. These findings call for effective sustainable interventions, including family and school-based hygienic education, to be strengthened to reduce the risk of infection among the susceptible population. Based on these findings, molecular techniques are recommended to explore the sources of infection. An early warning system based on a spatiotemporal model with risk factors is required to further improve the effectiveness and efficiency of cryptosporidiosis control in the future.

## Figures and Tables

**Table 1:** Characteristics of the participants in this study.

Variables	No. of Participants	%
Total	1637	100
Gender		
Male	864	52.8
Female	773	47.2
Age (years)		
3–6	863	52.7
7–9	774	47.3
Location (Township)		
A	330	20.2
B	242	14.8
C	324	19.8
D	741	45.3
Supplied with piped water		
Yes	1594	97.4
No	43	2.6
Presence of toilets at household		
Yes	1580	96.5
No	57	3.5
Raising livestock or poultry (e.g., cattle, sheep, chicken)		
Yes	756	46.2
No	881	53.8
Contact with pets (e.g., dog, cat)		
Yes	416	25.4
No	1221	74.6

**Table 2:** Univariable and multivariable analyses of risk factors associated with *Cryptosporidium* infection in this study (*n* = 1637).

Variables	Examined n	Infected n (‰)	cOR (95% Cl)	*p*	aOR (95% Cl)	*p*
Gender						
Male	864	8 (9)	0.713 (0.280–1.815)	0.476	0.713(0.278–1.828)	0.481
Female	773	10 (13)	1			
Age (years)						
3–6	863	14 (16)	3.175 (1.041–9.709)	0.032	3.072 (1.001–9.427)	0.050
7–9	774	4 (5)	1			
Location (Township)						
A	330	3 (9)	1.00	0.031	1	0.244
B	242	0 (0)	0.00	0.266	0.00	0.995
C	324	1 (3)	0.337 (0.035–3.261)	0.624	0.354 (0.036–3.438)	0.371
D	741	14 (19)	2.099 (0.599–7.354)	0.236	2.196 (0.624–7.734)	0.221
Supplied with piped water						
No	43	0 (0.0)	0.00	1.000		
Yes	1594	18 (11)	1			
Presence of toilets at household						
Yes	1580	17 (11)	0.609 (0.080–4.651)	0.473		
No	57	1 (18)	1			
Raising livestock or poultry						
Yes	756	7 (9)	0.739 (0.285–1.915)	0.533		
No	881	11 (12)	1			
Contact with pets (e.g., dog, cat)						
Yes	416	3 (7)	0.584 (0.168–2.028)	0.559		
No	1221	15 (12)	1			
Boiling water before drinking						
Yes	1361	15 (11)	1.014 (0.292–3.521)	1.000		
No	276	3 (11)	1			
Swimming in one month						
Yes	104	1 (10)	0.866 (0.114–6.579)	1.000		
No	1533	17 (11)	1			
Travelling in six months						
Yes	269	2 (7)	0.632 (0.145–2.770)	0.770		
No	1368	16 (12)	1			
Contact with water facilities in one month						
Yes	74	0 (0)	0.00	1.000		
No	1563	18 (12)	1			
Contact with a person with diarrhea in three months						
Yes	264	4 (15)	1.492 (0.488–4.566)	0.700		
No	1373	14 (10)	1			
Washing hands before eating and after defecation						
No	753	13 (17)	3.088 (1.096–8.703)	0.025	3.003 (1.060–8.511)	0.039
Yes	884	5 (6)	1			

**Table 3:** Details of spatial cluster of *Cryptosporidium* infection in Township D.

Cluster	Radius (km)	Number of Observed Cases	Number of Expected Cases	Population	RR	*p*
Primary	2.86	14	8.16	15602	4.220	0.025

## Supplementary Materials

The following are available online at http://www.mdpi.com/1660-4601/15/5/924/s1, Table S1: Details of the questionnaire in the study.

## Abbreviations

The following abbreviations are used in the manuscript:

OR	Odds Ratios
CI	Confidence Intervals
CDC	Center for Disease Control and Prevention
cOR	crude Odds Ratios
aOR	adjusted Odds Ratios
EBK	Empirical Bayesian Kriging
RR	Relative Risk
