# Supplementary material for: Risk Factors and Spatial Clusters of Cryptosporidium Infection among School-Age Children in a Rural Region of Eastern China
Source: Int J Environ Res Public Health. 2018 May 6;15(5):924. doi: 10.3390/ijerph15050924 (PMC5981963; doi:10.3390/ijerph15050924)
Supplement: Supplementary file 1 [file ijerph-15-00924-s001.pdf]

Supplementary Materials: Risk Factors and Spatial Clusters of Cryptosporidium Infection among School-Age Children in a Rural Region of Eastern China

Hao Zheng, Jianfeng He, Li Wang, Rong Zhang, Zhen Ding and Wenbiao Hu

Table S1. Details of questionnaire in the study.

Variables	Options
Demographic variables	
Name	
Gender	1. Male<br>2.Female
Age	
Address	
Environmental variables	
Supplied with piped water	1. Yes<br>2. No
Presence of toilets at household	1. Yes<br>2. No
Raising livestock or poultry	1. Yes<br>2. No
Personal characteristics	
Contact with pets (e.g., dog, cat)	1. Yes<br>2. No
Boiling water before drinking	1. Yes<br>2. No
Swimming in one month	1. Yes<br>2. No
Travelling in six months	1. Yes<br>2. No
Contact with water facilities in one month	1. Yes<br>2. No
Contact with a person with diarrhea in three months	1. Yes<br>2. No
Washing hands before eating and after defecation	1. Yes<br>2. No
Symtoms (e.g., diarrhea, nausea)	1. Yes<br>2. No

© 2018 by the authors. Licensee MDPI, Basel, Switzerland. This article is an open access article distributed under the terms and conditions of the Creative Commons Attribution (CC BY) license (http://creativecommons.org/licenses/by/4.0/).
